# Collagen extract obtained from Nile tilapia (*Oreochromis niloticus* L.) skin accelerates wound healing in rat model via up regulating VEGF, bFGF, and α-SMA genes expression

**DOI:** 10.1186/s12917-020-02566-2

**Published:** 2020-09-24

**Authors:** Zizy I. Elbialy, Ayman Atiba, Aml Abdelnaby, Ibrahim I. Al-Hawary, Ahmed Elsheshtawy, Hamed A. El-Serehy, Mohamed M. Abdel-Daim, Sabreen E. Fadl, Doaa H. Assar

**Affiliations:** 1 Fish processing and Biotechnology Department, Faculty of Aquatic and Fisheries Sciences, Kafrelsheikh University, Kafr el-Sheikh, Egypt; 2 Department of Surgery, Anesthesiology and Radiology, Faculty of Veterinary Medicine, Kafrelsheikh University, Kafr el-Sheikh, Egypt; 3 Department of Zoology, College of Science, King Saud University, P.O. Box 2455, 11451 Riyadh, Saudi Arabia; 4 Pharmacology Department, Faculty of Veterinary Medicine, Suez Canal University, 41522 Ismailia, Egypt; 5 Biochemistry Department, Faculty of Veterinary Medicine, Matrouh University, 51744 Matrouh, Egypt; 6 Clinical Pathology Department, Faculty of Veterinary Medicine, Kafrelsheikh University, Kafr el-Sheikh, Egypt

**Keywords:** Tilapia collagen, Wound healing, VEGF, bFGF, TGF-ß, α-SMA, IHC, Histopathology, Gene expression

## Abstract

**Background:**

Collagen is the most abundant structural protein in the mammalian connective tissue and represents approximately 30% of animal protein. The current study evaluated the potential capacity of collagen extract derived from Nile tilapia skin in improving the cutaneous wound healing in rats and investigated the underlying possible mechanisms. A rat model was used, and the experimental design included a control group (CG) and the tilapia collagen treated group (TCG). Full-thickness wounds were conducted on the back of all the rats under general anesthesia, then the tilapia collagen extract was applied topically on the wound area of TCG. Wound areas of the two experimental groups were measured on days 0, 3, 6, 9, 12, and 15 post-wounding. The stages of the wound granulation tissues were detected by histopathologic examination and the expression of vascular endothelial growth factor (VEGF), and transforming growth factor (TGF-ß1) were investigated using immunohistochemistry. Moreover, relative gene expression analysis of transforming growth factor-beta (TGF-ß1), basic fibroblast growth factor (bFGF), and alpha-smooth muscle actin (α-SMA) were quantified by real-time qPCR.

**Results:**

The histopathological assessment showed noticeable signs of skin healing in TCG compared to CG. Immunohistochemistry results revealed remarkable enhancement in the expression levels of VEGF and TGF-β1 in TCG. Furthermore, TCG exhibited marked upregulation in the VEGF, bFGF, and α-SMA genes expression. These findings suggested that the topical application of Nile tilapia collagen extract can promote the cutaneous wound healing process in rats, which could be attributed to its stimulating effect on recruiting and activating macrophages to produce chemotactic growth factors, fibroblast proliferation, and angiogenesis.

**Conclusions:**

The collagen extract could, therefore, be a potential biomaterial for cutaneous wound healing therapeutics.

## Background

The wound is a clinical surgical entity that requires medical attention. Wound healing is a multi-process including 4 interdependently definite stages; hemostasis, inflammatory response, tissue proliferation, and remodeling [1]. The hemostasis, blood clotting, the stage starts directly after the injury and it is characterized by blood platelet aggregation, which subsequently produces various chemotactic factors, for instance transforming growth factor-beta 1&2 (TGF-ß1) & (TGF- ß2), and platelet-derived growth factors (PDGF). Furthermore, inflammatory cells (microphages, lymphocytes, and macrophages) are recruited to protect the wound from infection. Besides their crucial role in protecting the wound, the inflammatory cells along with epidermal and dermal cells produce some mediators. These mediators function by regulating and stimulating growth and migration of smooth muscle cells, keratinocytes, and fibroblasts within the wound area, which plays an essential role in the wound healing process [2].

Collagen is the most abundant structural protein in the mammalian connective tissue and represents approximately 30% of animal protein. Mammalian collagens, porcine, and bovine, have been widely utilized for many years, however, outbreaks of Foot and Mouth Disease (FMD) and Animal Prion Diseases in the last era have paid attention to their safety and therefore restricted their use. Thus, aquatic organisms for instance fish, starfish, sponges, and jellyfish have been recently evaluated as a safe easily accessible alternative for mammalian collagen [3].

Besides their crucial role as a safe alternative for mammalian collagen, fish collagen peptides possess various biological benefits such as immune-modulatory and anti-inflammatory activities [4], antimicrobial activity [5] and improve wound healing [6, 7]. Marine collagen has been isolated from different marine and freshwater fish species including; Chum Salmon (*Oncorhynchus keta*) [8], Spanish mackerel (*Scomberomorous niphonius*) [9], Hybrid Sturgeon [10], Bigeye snapper (*Priacanthus macracanthus*) [11], Silver carp (*Hypophthalmichthys molitrix*) [12], Sheepshead seabream (*Archosargus probatocephalus*) [13], Black drum (*Pogonia cromis*) [13], Bigeye snapper (*Priacanthus tayenus*) [14], and many other fish species.

Nile tilapia, *Oreochromis niloticus*, is considered one of the most extensively cultured species worldwide with a global production of 6,5 million tones in 2018 [15]. The skin of tilapia constitutes an enormous amount of non-edible by-product, however, it could be used as an excellent substrate for the production of collagen peptides. Studies suggested that using tilapia skin could provide more than 40% dry weight yield of collagen. Moreover, the use of tilapia skin in the production of collagen peptides provides a safe product without any ethical or religious concerns [16]. Hence, some investigations have recommended the use of tilapia skin as a biomaterial for dressing the wound healing [17, 18]. Nevertheless, little is known about the potential mechanisms of tilapia derived collagen in improving wound healing. Thus, the current study aimed to clarify the potential healing capacity of tilapia skin-derived collagen in rat wound healing and the underlying potential mechanisms.

## Results

### The results of functional group (FTIR) analysis

**Fig. 1:**
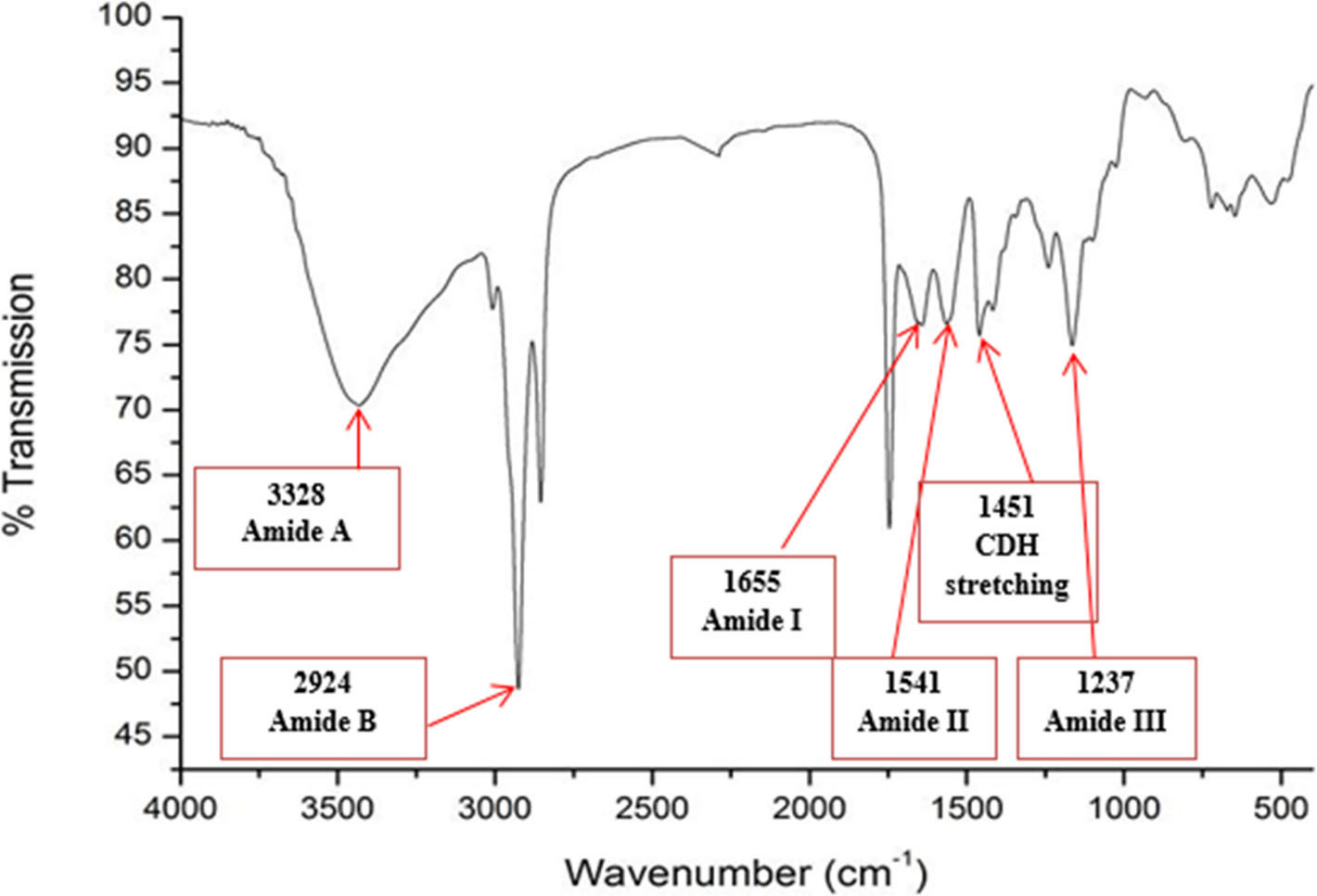
FTIR spectrum of collagen extracted from tilapia skin

The FTIR spectra of collagen extracted from the skin of tilapia shown in Fig. 1. The obvious peaks at 3328 cm-1 and 2924 cm-1 were typical characteristic amide A and B bands, respectively. The band at 1451 cm-1 was attributed to the CDH stretching vibrations. The characteristic absorption peaks of amide I, II, and III bands of polypeptides were at 1655, 1541, and 1237 cm-1, respectively.

### Wound area

**Fig. 2:**
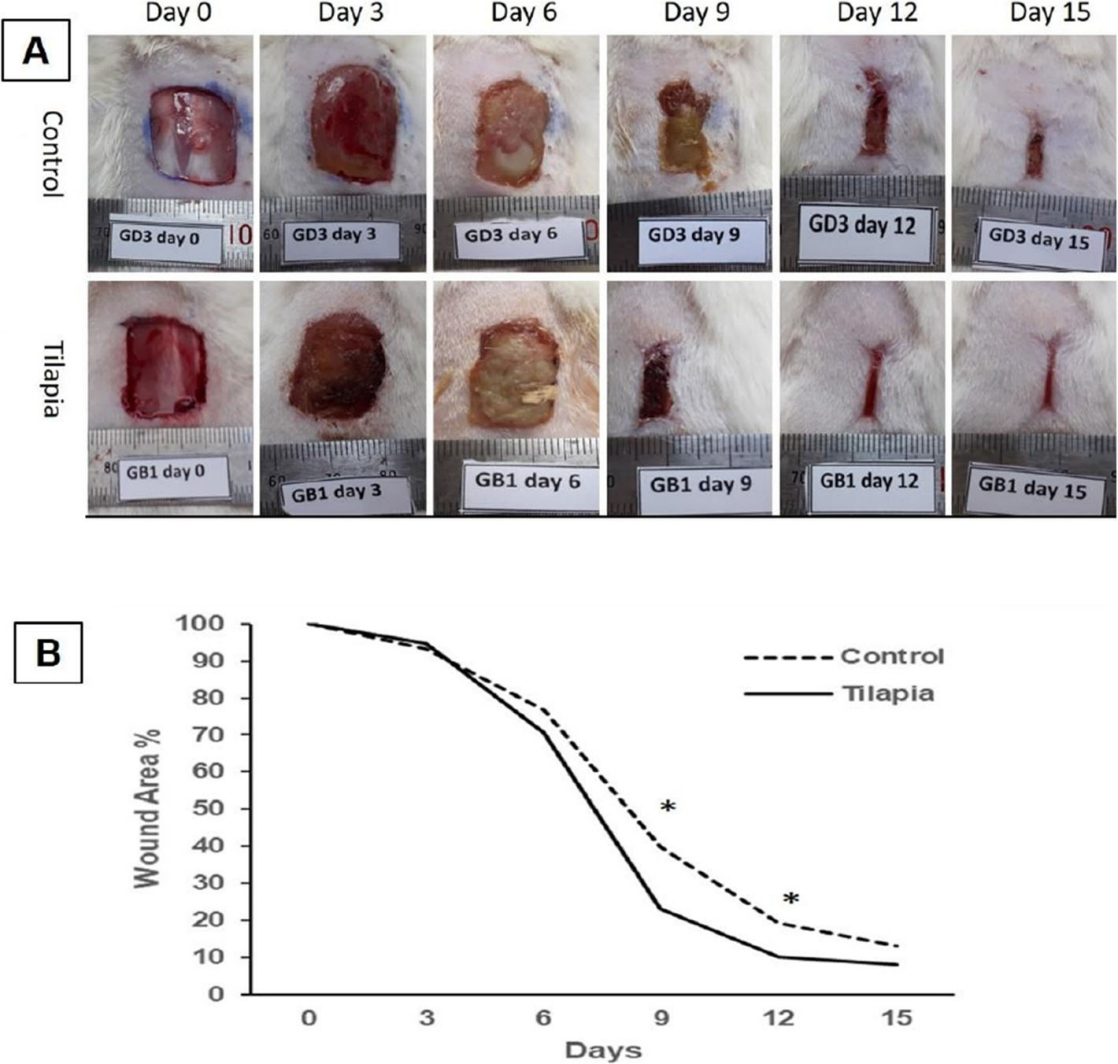
**a** Effect of topical application of Tilapia collagen extract on wound area contraction in TCG: Tilapia collagen group compared to the CG :control group. **b** The percentage of wound area on days 0,3,6,9,12 and 15 post wounding as compared with wound closure in control group. The wound closure rate was expressed as the percentile of wound area compared with that on post wounding day 0 (100%).Values are mean ± SE

The impact of the tilapia collagen extract on wound area is presented in (Fig. 2). The contraction of the wound area in TCG was significantly promoted compared to the CG. Moreover, the wound area on days 9, 12, and 15 post-wounding in TCG displayed smaller wound areas than the CG (Fig. 2a). Wound contraction in TCG was accelerated significantly (*P* ˂ 0.05) on days 9 and 12 post wounding in which the wound sites tended toward closure in the TCG and the adjacent skin became smoother than the CG (Fig. 2b).

### Histopathological examination

**Fig. 3:**
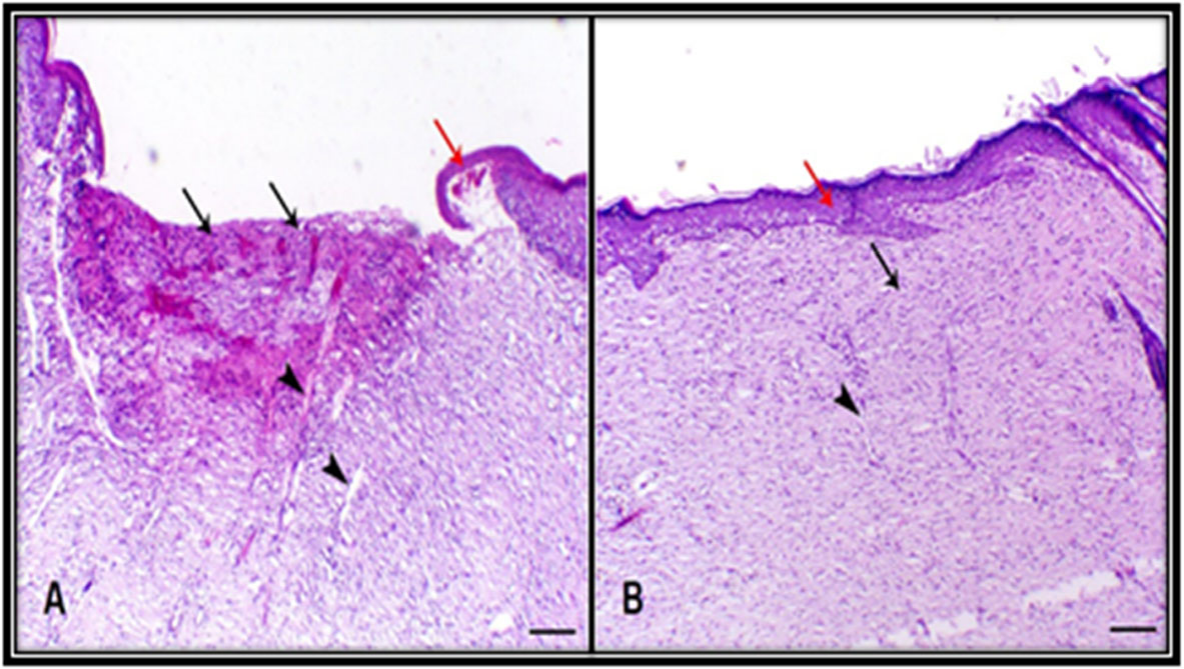
Histological examination at day 15 post-wounding. **a** Skin of control group animal showing delayed skin healing (black arrows) with marginal epithelial tongue formation (red arrow) and still granulation tissue within the connective tissue (arrowhead indicates angiogenesis), **b** Skin of tilapia collagen group animal showing marked skin healing accompanied with marked epithelization (red arrow) and remodeling of connective tissue (red arrow) (arrowhead indicates angiogenesis) H&E, bar = 200 µm

Histological examination on day 15 post-wounding revealed that the skin of CG had delayed skin healing with marginal epithelial tongue formation with remaining granulation tissue within the connective tissue, which indicates angiogenesis. However, TCG had marked skin healing accompanied by a marked reduction in the dermal tissue with remarkable epithelization and remodeling of connective tissue (Fig. 3) (Table 1).

**Table 1:** Semi-quantitative scoring of skin healing of different treated groups

Groups	Necrosis	Granulation tissue	Connective tissue remodeling	Epithelization	Inflammatory cells infiltration
A	+	++	++	++	+
B	-	+	++++	++++	+

Histological scale: - Indicates absence of lesions, + indicates mild degree, ++ indicates moderate degree, +++ indicates marked focal degree and ++++ indicates marked diffuse degree

### Immunohistochemistry examination

**Fig. 4:**
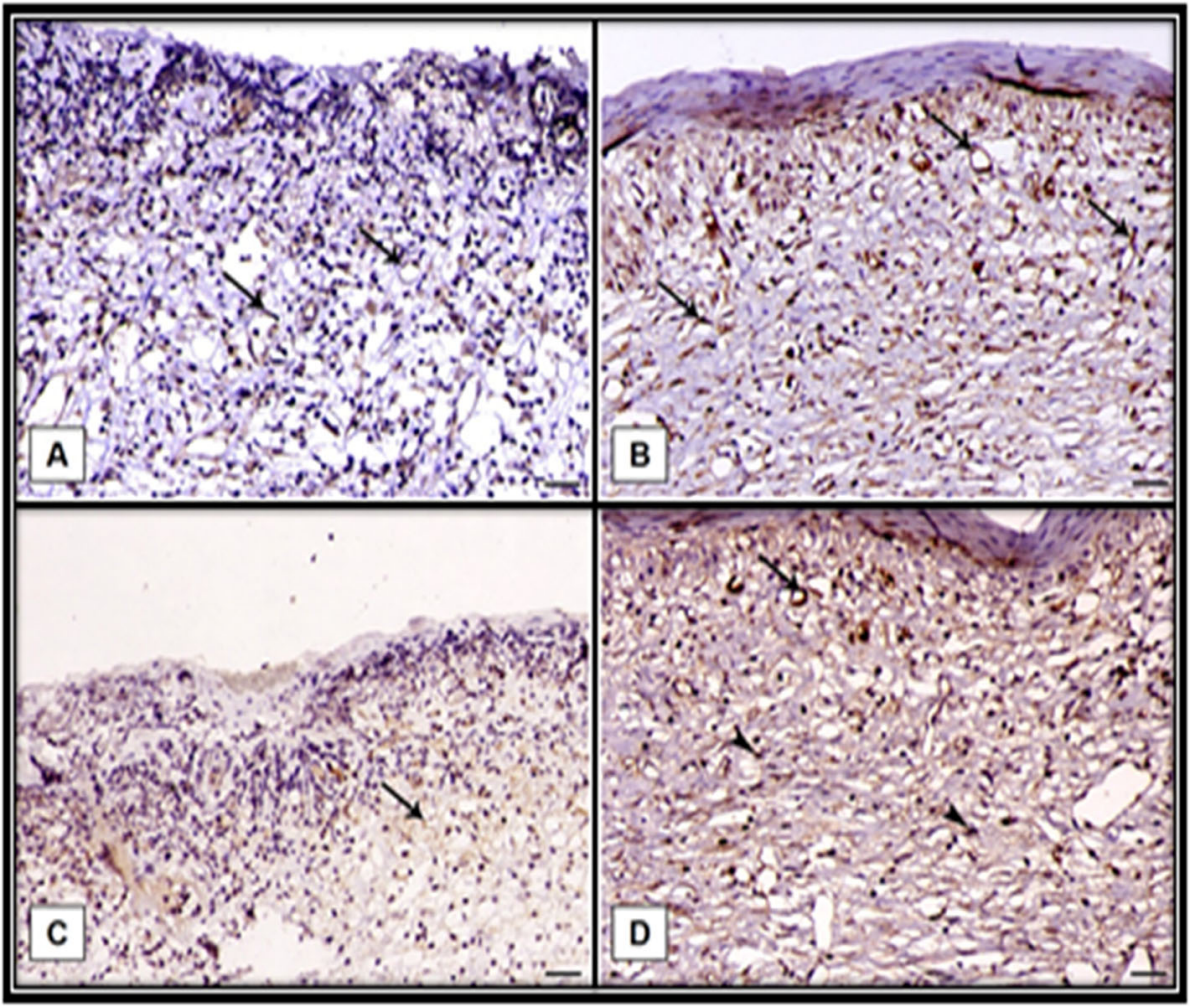
Immunohistochemistry results of *VEGF* : Vascular endothelial growth factor and *TGF-β1*:Transforming growth factor *β1*. **a** Control group showed mild *VEGF* expression of within the endothelial cells of the granulation tissue (arrows). **b** Tilapia group showed marked increase *VEGF* expression of within the endothelial cells of the mature granulation tissue underlying the epidermis (arrows). **c** Control group showing mild expression of *TGF-β1* within the endothelial cells of the granulation tissue (arrows). **d** Tilapia group showed marked *TGF-β1* expression either within the endothelial cells of blood capillaries (arrow) or fibroblastic cells (arrowheads), X200, bar = 40 µm

Angiogenesis plays a key role during wound healing. The renewed blood vessel provides nutrition and oxygen supplements to the wound area and assists in the formation of granulation tissue. To examine the effect of the tilapia collagen on angiogenesis, fibroblast proliferation, and collagen deposition, immunohistochemical staining (IHC) was used to investigate the VEGF and TGF-β1 expression in skin tissue on day 15 post-wounding. A noticeable increase in both VEGF and TGF-β1 in the TCG was observed (Table 2). The expression profiles of VEGF and TGF-β1 are presented in (Fig. 4). There was a markedly higher expression in both VEGF and TGF-β1 in the TCG either within the endothelial cells of blood capillaries or fibroblastic cells (Fig. 4b and d). However, CG revealed mild expression in both VEGF and TGF-β1within the endothelial cells of the granulation tissue (Fig. 4a and c). These results illustrated the significant change in the expression of VEGF and TGF-β1 upon the application of tilapia collagen extract on the wound.

**Table 2:** IHC results of rat skin expressed as mean ± SD

Groups	VEGF	TGFβ1
Control	35.825 ± 5.2	40.975 ± 1.56
Tilapia collagen	63.425 ± 4.4*	71.95 ± 2.79*

*IHS* Immunohistochemical staining, *CG* Control group, *TCG* Tilapia collagen group

Values are expressed as mean ± SE from triplicate groups (*n =* 06). Bars with an asterisk are significantly different from those of control group (*P* < 0.05)

### Gene expression

**Fig. 5:**
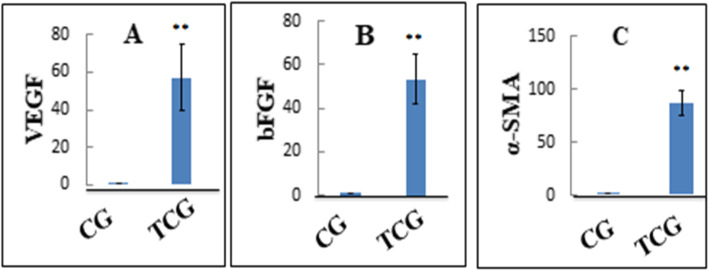
Relative gene expression of *VEGF*: Vascular endothelial growth factor, *bFGF*: basic fibroblast growth factor, and α-smooth muscle actin: *α-SMA*: genes in wounded rats of CG: control group and TCG: tilapia collagen treated group. Values are expressed as mean ± SE from triplicate groups (*n =* 06). Bars with an asterisk are significantly different from those of control group (*P* < 0.05)

To explore the potential molecular mechanisms of tilapia collagen extract impact on wound healing, we assessed the relative expression of TGF-β1, bFGF, and α-SMA genes in the wound tissue. The relative expression of TGF-β1, bFGF, and α-SMA genes was markedly upregulated in TCG as compared to CG (Fig. 5).

## Discussion

The results of the FTIR spectra of collagen extracted from the skin of tilapia are compatible with Zhang et al. [19], amide A bands appeared in the range 3292–3315 cm-1 proved that N–H group of peptides is involved in hydrogen bonding. Amide I band is observed at 1656 cm-1. Amide II band of collagen at 1538–1548 cm-1 while amide III band is found in the range 1232–1238 cm-1. Overall FTIR represents the characteristic amino acids and intact collagen molecule [20]. Also, Chen et al. [21] analyzed the FTIR spectra of ASC, which was extracted from the skin of tilapia and matched well with those of collagen extracted in our study. These results indicated that the extracted collagen was of type I collagen and maintained their triple-helical structure [20].

Wound healing is an integrated and dynamic process with an orchestrated cascade of events that requires a complicated interaction among injured skin cells, immune cells, cytokines, growth factors, and tissue sequences such as cellular migration, proliferation, angiogenic responses, and wound tissue fibroplasia [22]. Multiple growth factors including; transforming growth factor-beta1 (TGF-β1), fibroblast growth factor (FGF), and vascular endothelial growth factor (VEGF) are incorporated in the process of healing by promoting cell proliferation through activation of numerous steps as angiogenesis, re-epithelialization, and formation of the extracellular matrix (ECM) [2, 23, 24]. FGF triggers the proliferation and migration of fibroblasts, angiogenesis, epithelialization, and ECM production in wound healing [24, 25]. Moreover, TGF-β1 is essential for the synthesis of ECM by the activated fibroblast and the differentiated myofibroblast [26–28]. Angiogenesis is a crucial step to ensure a sufficient supply of nutrients, oxygen, and immune cells to the stroma and it is precisely controlled by the innate immune response [29]. Furthermore, responding to anoxia, angiogenic factors, such as platelet-derived growth factor (PDGF), VEGF, and bFGF, trigger the genesis of new blood supply. Additionally, the contractile microfilament bundles “myofibroblasts”, formed during the healing process by fibroblasts, are essential for the contraction of the wound [2, 30, 31]. The entirely differentiated myofibroblast can be recognized by the expression of α-smooth muscle actin (α-SMA) [2, 31, 32]. Keratinocytes are also considered key cells involved in skin wound healing and contraction. The proliferation and differentiation of keratinocytes play essential roles in wound re-epithelialization [33]. Fibroblasts and keratinocytes produce new collagen fibers, which form a transient ECM, which is then remodeled and strengthened as a consequence of matrix metalloproteinases activity regulated with the activity of tissue inhibitors of metalloproteinases [34, 35].

The current study highlighted the key role of tilapia derived collagen in wound contraction and healing process in rats utilizing histopathology, immunohistochemistry, and gene expression analysis. The study revealed the potential capacity of tilapia collagen in accelerating the full-thickness skin wound healing in rats in agreement with several in *vivo* and in *vitro* studies [17, 18, 36]. For instance, Chen, Gao [36] reported that tilapia collagen treatment leads to accelerate epithelization and healing process by promoting keratinocyte proliferation and differentiation. Our data revealed that TGF-β1, IHC, was markedly enhanced and TGF-β1, b-FGF, and α-SMA genes expressions were strongly upregulated in TCG, indicating the enhanced proliferation of both fibroblast and myofibroblast, and increased ECM production in TCG than CG. Our findings are inconsistent with Wang, Xu [37] who examined the impact of collagen peptides derived from chum salmon on wound healing of cesarean sectioned rats. The same trend was reported by Hu, Yang [17] who detected the wound healing acceleration property of collagen peptides obtained from Nile tilapia skin in rabbits.

Angiogenesis is a vital process for the maintenance of granulation tissue and hastening wound healing and it is induced by several angiogenic factors such as bFGF and VEGF [38, 39]. Our findings illustrated that VEGF, IHC, and bFGF gene expression levels in wound tissue were significantly higher in TCG than CG, implying that the tilapia derived collagen has a potent ability to enhance the production of these angiogenic factors. The higher expression of such factors is remarkably implicated in promoting the wound healing process by controlling the inflammatory reaction and enhancing angiogenesis and collagen deposition with a marked reduction in the dermal tissue width and improved epithelization rate in TCG, referring to a faster wound closure rate in TCG than the CG.

## Conclusions

It is concluded that the topical applicated tilapia skin collagen extract enhanced the cutaneous wound healing in the rat model. The improved wound healing could be linked to its stimulating effect on recruiting and activating macrophages to produce chemotactic growth factors, fibroblast proliferation, and angiogenesis, upon the upregulation of TGF-β1, bFGF, and α-SMA genes expression and enhanced TGF-β1 and VEGF expression.

## Methods

### Ethical statement

Ethical approval for the experimental procedures of the study was obtained from the Animal Ethics Committee at Kafrelsheikh University, Egypt.

### Tilapia skin collagen extraction

The fish were obtained from a private farm at Kafrelsheikh Governorate, Egypt. Nile tilapia skin collagen was extracted by the preparation of acid and pepsin-solubilized collagen according to the Noitup method described by Potaros, Raksakulthai [40]. In brief, fish were euthanized with an overdose of buffered MS-222 (one fish at the time) in a separate container (200 mg/L MS-222 + 400 mg/L sodium bicarbonate). The skin of *O. niloticus* was separated by scalpel and cut into small pieces (0.5 × 0.5 cm) with scissors. The non-collagenous proteins were removed from the cut skin where the skin was suspended in 20 volumes NaOH (0.1 N) at pH 12 and stirred for 4 hr. Then the cut skin was washed thoroughly with cold distilled water (less than 10 °C) until a neutral pH was obtained. After that acid-solubilized collagen was produced where the washed skin was extracted with 70 volumes of acetic acid (0.5 M) at 4–6 °C for 24 hr. The extracted acid-solubilized collagen was centrifuged for 60 min at 30,000 × g and collected the supernatant. Meanwhile, the precipitate was re-extracted two times. After that, the collected supernatant was salted out by adding NaCl (0.9 M) and centrifuged at 30,000 × g for 60 m. The precipitate was dissolved in 20 volumes of 0.5 M acetic acid, then dialyzed against 0.1M acetic acid and deionized water in a dialysis membrane (Spectra/ por mwco 12–14,000 RC membrane, Spectrum Laboratories, Rancho Dominguez, CA, USA.). Apart from the obtained gel form extracted collagen was lyophilized and measured with Fourier transform infrared spectroscopy (FTIR) spectrum measurement.

### Fourier transform infrared spectroscopy (FTIR) spectrum measurement

Collagen FTIR spectrum measurement was conducted using FTIR (Fourier-transform infrared spectroscopy, Thermo Scientific Nicolet iS5, MA, USA). The lyophilized collagen powder was mixed with KBr (Sigma-Aldrich, St. Louis, MO), then their mixture was pressed and subjected to analysis [41]. The main FTIR parameters’ settings were the 4 cm-1 resolution and scan number of 64 times; the transmission spectrum within 450–4000 cm-1 was recorded.

### Animals

Ten weeks old male Wistar rats (110 ± 10 g) were obtained from the Animal House Colony Tanta Centre, Egypt. The lab animals (16 rats) were randomly assigned into 2 groups (8 rats per each group); the control group (CG), did not receive any treatment and tilapia collagen treated group (TCG), treated with gel form collagen extract. In separate plastic cages, the laboratory animals were kept with fed (Al Wadi, Egypt) and water ad libitum with a 12 h dark/light cycle and temperature 25 ± 2˚C. Where animals were acclimatized for one week before the beginning of the experiment.

### Wound creation and wound size assessment

**Fig. 6:**
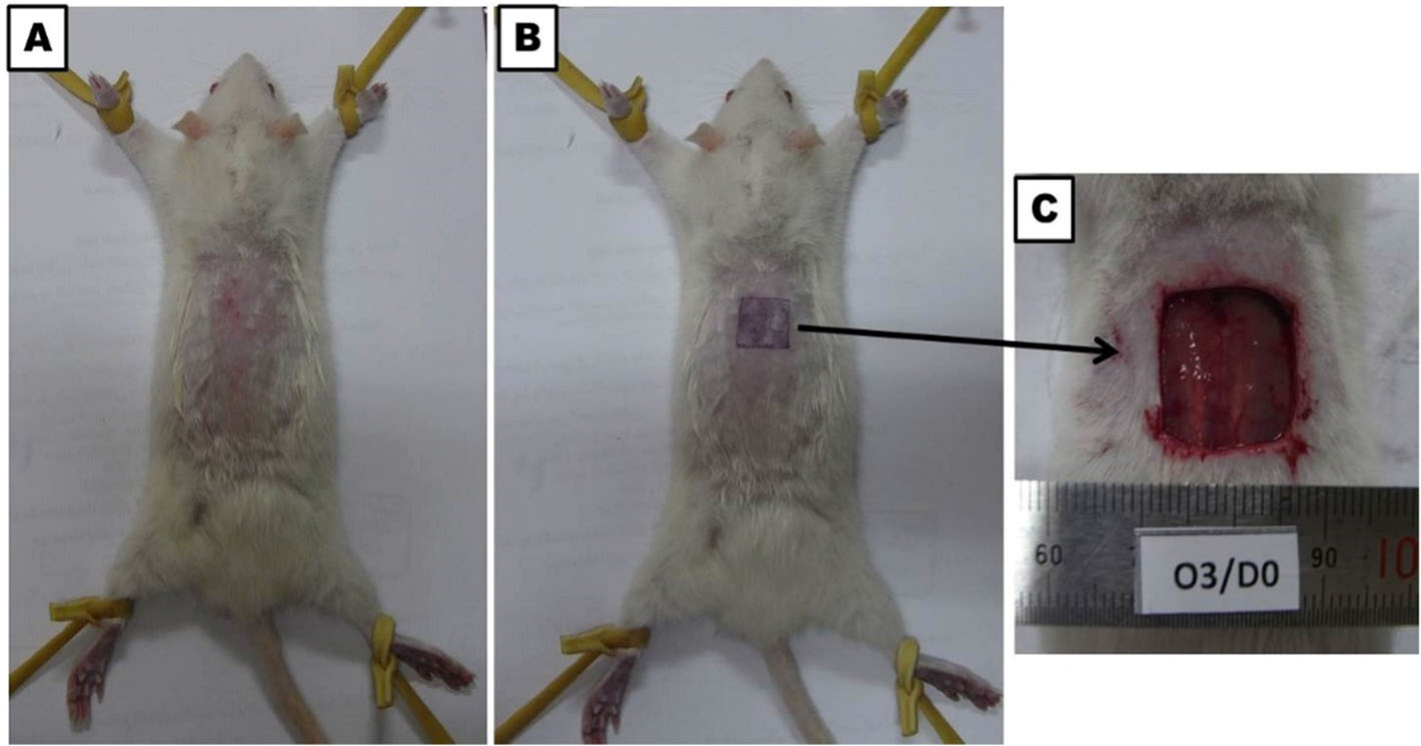
**a** Preparation to perform full thickness skin incision by clipping back hair of the rat then disinfection by ethyl-alcohol. **b** Putting marker for making definite skin incision. **c** Photo represent day of wounding (day 0)

The ketamine-xylazine combination (ketamine 70 mg/kg and xylazine 7 mg/kg) were used intraperitoneal injection (IP) for anesthetization of the laboratory animals. The rats’ back hair was shaved and disinfected by 70% ethanol. Next, full-thickness skin wound excision measuring 1.5 × 1.5 cm was performed on the back of each rat according to Atiba et al. [25] (Fig. 6). The wound without suture left opened where it was covered by wound dressing (Band-Aid Advanced healing (Johnson & Johnson)) and changed every two days on days 0, 3, 6, 9, and 12 post-wounding. Band-aid dressing was fixed by medical tape. Wound dressing was applied under anesthesia. In the control group, no treatment was used only changing the dressing. Meanwhile, in the TCG group, the gel form collagen extract was applied every time the dressing was changed.

The wounds were photographed and measured on days 0, 3, 6, 9, 12, and 15 post-wounding (Fig. 6). The images were then converted into the TIF Format using Adobe Photoshop software. The size area of wounds was measured by the ImageJ-NIH software (available online at; http://www.rsb.info.nih.gov/ij). The change of the wound size was demonstrated as a percentage of the initial wound size (day 0:100%).

**Fig. 7:**
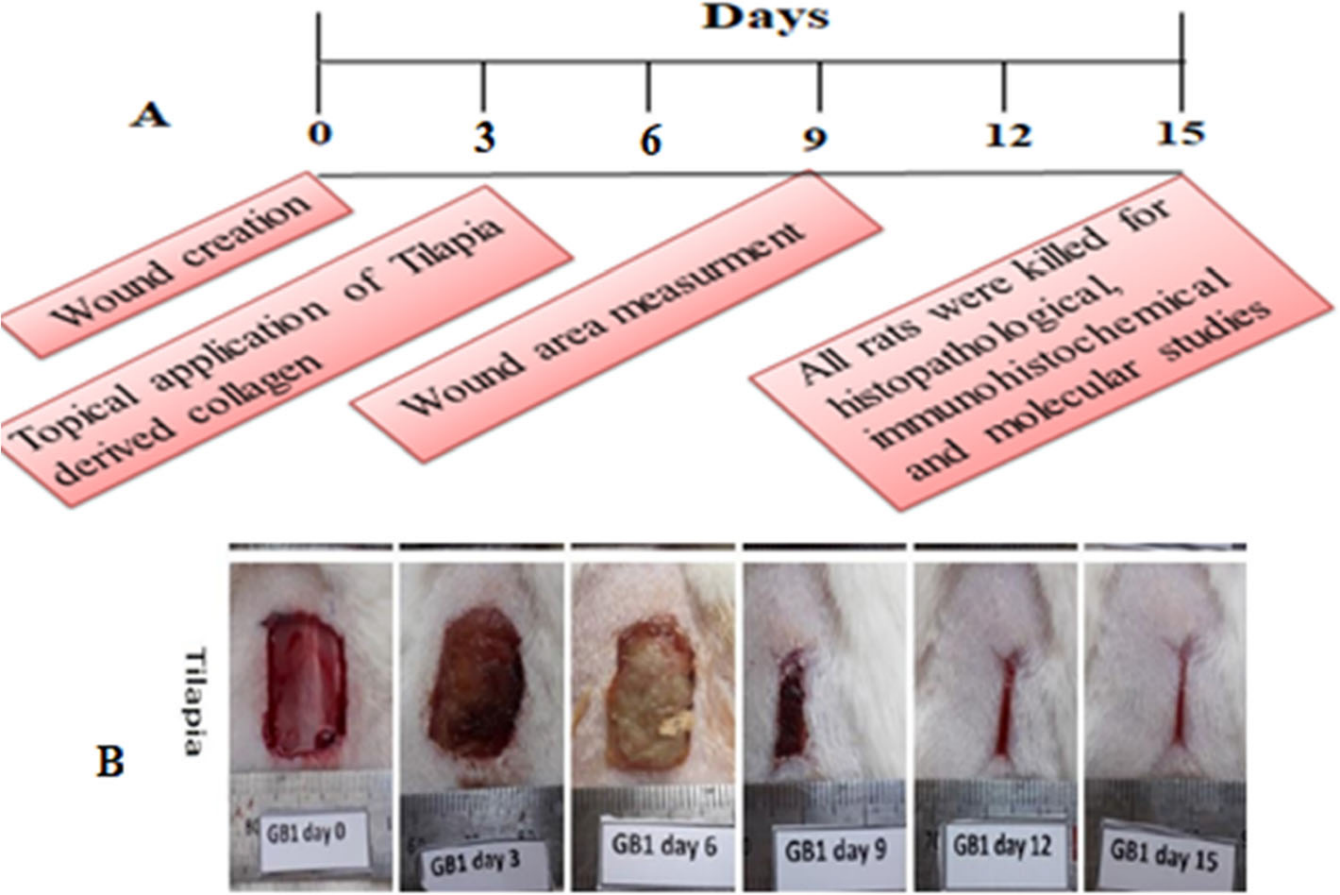
Experimental scheme of this study. **a** Schedule time of wounding, wound area measurement, Tilapia collagen extract administration and sampling. **b** Wound dressing with Band –Aid, wound area photographed and measurements

Experimental scheme of this study; **(A)** Schedule time of wounding, wound area measurement, Tilapia collagen extract administration, and sampling. **(B)** Wound dressing with Band–Aid, wound area photographed, and measurements is portrayed in (Fig. 7).

### Tilapia collagen application

Tilapia derived collagen extract in gel form was applied topically on the wound area of the tilapia treated collagen group, while the control group did not receive any treatment. Collagen was applied topically to wound area on days 0, 3, 6, 9, and 12 post-wounding with wound dressing and measuring the area of wound each time.

The dressing was changed to both groups in the mentioned days of the experiment with inspection of the wound healing degree.

### Tissue samples collection

On day 15 post-wounding, rats were sacrified by intraperitoneal injection of an overdose of pentobarbital anesthesia (300 mg/kg). The whole wound including a periphery of about 5 mm of unwounded skin area was excised. Excised skin samples were divided into two parts; one was fixed for 48 hrs. in a 10% formalin buffered solution (pH 7.4) and embedded in paraffin (used for histopathology and immunohistochemistry). The other part was collected in 2 mL sterile microcentrifuge tubes, then shocked in liquid nitrogen and then stored at -80ºC till the analysis (used for gene expression analysis). The investigators were not blinded during data collection. Blinding was used during analysis. Computational analysis was not performed blinded.

### The rats after the end of the study

All rats were sacrified by intraperitoneal injection of an overdose of pentobarbital anesthesia (300 mg/kg). The sacrified rats, as well as remnants of samples and bedding material, were buried in the strict hygienically controlled properly constructed burial pit.

### Histopathological examination

Skin wound tissues sections of 4 µm thickness were perpendicularly cut into the wound and stained with hematoxylin and eosin. The following parameters were individually assessed; the degree of necrosis, granulation tissue, connective tissue remodeling, degree of epithelialization, and inflammatory cell infiltration. The scores of wound granulation tissue for different treated groups were semi-quantitated as illustrated in Table 1. Histopathology slides were graded using a modified 0 to 4 Ehrlich and Hunt numerical scale and validated on a scale 1–5, with 1 indicating necrosis, 2 indicating inflammatory cell infiltration, 3 indicating connective tissue remodeling, 4 indicating epithelization, and 5 indicating inflammatory cell infiltration. We applied a five-point scale to evaluate them (-ve: Indicates absence of lesions, +: indicates mild degree, ++: Indicates moderate degree, +++: Indicates marked focal degree and ++++: Indicates marked diffuse degree) as previously described by [42].

### Immunohistochemistry examination

The immunohistochemical staining procedures were performed according to Saber et al. [43]. Skin tissues embedded in paraffin were sectioned (4 mm thick), dewaxed, and immersed in a citrate buffer (0.05 M, pH 6.8), for antigen retrieval. Then, these sections were treated with 0.3% H_2_O_2_ and protein block. Next, they were incubated with polyclonal rabbit TGF-β1 (Cat. No. 1-TR062-07, BIOCYC GmbH, & Co. KG, Germany) and polyclonal rabbit VEGF (Cat. No. AR483-5R, BioGenix, Netherland). After washing with PBS, they were incubated for 30 min at room temperature with a goat anti-rabbit secondary antibody (cat. no. K4003, EnVision + System- HRP Labelled Polymer; Dako). Slides were then visualized with DAB kit and finally stained with, a counterstain, Mayer’s hematoxylin, and assessed under the light microscope. The staining intensity was evaluated and presented as a percentage of positive cells in about 8 high power fields.

### RNA extraction

RNA was extracted from all collected samples using TriZol reagent (iNtRON Biotechnology) following the manufacture protocol. The quality and concentration of the extracted RNA were assessed using a nanodrop spectrophotometer (BioDrop). Samples with ODA260/ A280 from 1.8 to 2.0 were used. The integrity of all extracted RNA was confirmed by 1.5% ethidium bromide-stained agarose gel (Sigma, Germany) in 1x Tris-acetate-EDTA buffer, pH 8.0. The gel image was visualized using UV transilluminator (azure c200).

### cDNA synthesis and real-time qPCR

RT-qPCR analysis of relative mRNA expression of TGF-β1, bFGF, αSMA, and glyceraldehyde-3-phosphate dehydrogenase (GAPDH) as the housekeeping gene was performed using rat-specific primers Table 3 [32, 44–46]. cDNA synthesis from 2 µg of the total RNA was performed using the Intron-Power cDNA synthesis kit (iNtRON Biotechnology) following the manufacturer’s protocol. Then, the cDNA was used as the template for RT-qPCR.

**Table 3:** Primer sequence of selected genes used in rtPCR analysis

Gene	Primer sequence 5’-3’	Gene bank accession number	Annealing temperature	Reference
GAPDH	5'-CAGCAATGCATCCTGCAC-3' 5'-GAGTTGCTGTTGAAGTCACAGG-3'	XM_017592435.1	60ºC	Nakahara et al. [44]
VEGF	5'-AGGCTGCACCCACGACAGAA-3' 5'-CTTTGGTCTGCATTCACATC-3'	NM_001110333.2	55ºC	Hu et al. [45]
bFGF	5'-GTCAAACTACAGCTCCAAGC-3' 5'-TTTATACTGCCCAGTTCGTT-3'	NM_019305.2	50 ºC	Zhu et al. [46]
αSMA	5’-CGATAGAACACGGCATCATC-3’ 5’-CATCAGGCAGTTCGTAGCTC-3’	NM_031004.2	58 ºC	Reza Ghassemifar et al. [32]

*VEGF* Vascular endothelial growth factor, *bFGF* Basic fibroblast growth factor, *α-SMA* α-smooth muscle actin

Relative quantitative RT-PCR was performed to all the examined samples in triplicate using SYBR green in the Mx3005P Real-time PCR (Agilent, USA).

The relative changes in gene expression were calculated using threshold cycle (CT) values that were first normalized to those of the Albino Rat (*Rattus norvegicus*) GAPDH house-keeping gene and using ΔCT value of control samples as calibrator using the 2^−ΔΔCT^ method adapted from Livak and Schmittgen [47].

### Statistical analysis

The data are illustrated as the mean ± standard error (SE). Data were compared by one-way analysis of variance (ANOVA) followed by post-hoc t-test. The test was conducted for comparing each group with and without tilapia collagen treatment in wound area rate and relative gene expressions of TGF-β1, bFGF, αSMA. Differences among the groups were considered statistically significant at *p-*value < 0.05 and indicated with an asterisk (**).

## Data Availability

The datasets used and/or analyzed during the current study are available from corresponding author on reasonable request.

## Abbreviations

CG: Control group
DNA: Deoxyribonucleic acid
ECM: Extracellular matrix
FGF: Fibroblast growth factor
GAPDH: Glyceraldehyde-3-phosphate dehydrogenase
IP: Intraperitoneal injection
Q-PCR: Real-Time Polymerase Chain Reaction
PDGF: Platelet-derived growth factor
RNA: Ribonucleic acid
α-SMA: α-smooth muscle actin
TCG: Tilapia collagen treated group
TGF-β1: Transforming growth factor-beta1
VEGF: Vascular endothelial growth factor

## References

[1] Stadelmann WK, Digenis AG, Tobin GR. Physiology and healing dynamics of chronic cutaneous wounds. The American Journal of Surgery. 1998;176(2):26S-38S. 10.1016/s0002-9610(98)00183-4 9777970

[2] Darby IA, Laverdet B, Bonté F, Desmoulière A (2014). Fibroblasts and myofibroblasts in wound healing. Clin Cosmet Investig Dermatol.

[3] Addad S, Exposito JY, Faye C, Ricard-Blum S, Lethias C (2011). Isolation, characterization and biological evaluation of jellyfish collagen for use in biomedical applications. Marine Drugs.

[4] Azuma K, Osaki T, Tsuka T, Imagawa T, Okamoto Y, Minami S (2014). Effects of fish scale collagen peptide on an experimental ulcerative colitis mouse model. Pharma Nutr..

[5] Ennaas N, Hammami R, Gomaa A, Bédard F, Biron É, Subirade M, Beaulieu L, Fliss I. Collagencin, an antibacterial peptide from fish collagen: Activity, structure and interaction dynamics with membrane. Biochemical and biophysical research communications. 2016 Apr 29;473(2):642–7. 10.1016/j.bbrc.2016.03.121 27038545

[6] Yang T, Zhang K, Li B, Hou H (2018). Effects of oral administration of peptides with low molecular weight from Alaska Pollock (Theragra chalcogramma) on cutaneous wound healing. J Funct Foods..

[7] Zhang Z, Wang J, Ding Y, Dai X, Li Y (2011). Oral administration of marine collagen peptides from Chum Salmon skin enhances cutaneous wound healing and angiogenesis in rats. J Sci Food Agric.

[8] Gbogouri GA, Linder M, Fanni J, Parmentier M (2004). Influence of hydrolysis degree on the functional properties of salmon byproducts hydrolysates. J Food Sci.

[9] Li ZR, Wang B, Chi CF, Zhang QH, Gong YD, Tang JJ, Luo HY, Ding GF. Isolation and characterization of acid soluble collagens and pepsin soluble collagens from the skin and bone of Spanish mackerel (Scomberomorous niphonius). Food hydrocolloids. 2013 May 1;31(1):103 – 13.

[10] Wei P, Zheng H, Shi Z, Li D, Xiang Y (2019). Isolation and Characterization of Acid-soluble Collagen and Pepsin-soluble Collagen from the Skin of Hybrid Sturgeon. J Wuhan Univ Technology-Mater Sci Ed..

[11] Jongjareonrak A, Benjakul S, Visessanguan W, Tanaka M. Isolation and characterization of collagen from bigeye snapper (Priacanthus macracanthus) skin. J Sci Food Agric. 2005 May;85(7):1203–10.

[12] Rodziewicz-Motowidło S, Śladewska A, Mulkiewicz E, Kołodziejczyk A, Aleksandrowicz A, Miszkiewicz J, Stepnowski P (2008). Isolation and characterization of a thermally stable collagen preparation from the outer skin of the silver carp Hypophthalmichthys molitrix. Aquaculture..

[13] Ogawa M, Portier RJ, Moody MW, Bell J, Schexnayder MA, Losso JN (2004). Biochemical properties of bone and scale collagens isolated from the subtropical fish black drum (Pogonia cromis) and sheepshead seabream (Archosargus probatocephalus). Food Chem..

[14] Benjakul S, Thiansilakul Y, Visessanguan W, Roytrakul S, Kishimura H, Prodpran T, Meesane J. Extraction and characterisation of pepsin-solubilised collagens from the skin of bigeye snapper (Priacanthus tayenus and Priacanthus macracanthus). Journal of the Science of Food Agric. 2010;90(1):132–8. 10.1002/jsfa.3795 20355023

[15] FAO, *Global Aquaculture Production.* 1950*–2016*. 2019, Food and Agriculture Organization of the United Nations: Rome, Italy.

[16] Mei F, Liu J, Wu J, Duan Z, Chen M, Meng K, Chen S, Shen X, Xia G, Zhao M (2020). Collagen peptides isolated from salmo salar and tilapia nilotica skin accelerate wound healing by altering cutaneous microbiome colonization via upregulated NOD2 and BD14. J Agric Food Chem..

[17] Hu Z, Yang P, Zhou C, Li S, Hong P (2017). Marine collagen peptides from the skin of Nile Tilapia (Oreochromis niloticus): Characterization and wound healing evaluation. Marine Drugs.

[18] Zhou T (2016). Electrospun tilapia collagen nanofibers accelerating wound healing via inducing keratinocytes proliferation and differentiation. Colloids Surf B.

[19] Zhang Q, Wang Q, Lv S, Lu J, Jiang S, Regenstein JM, Lin L (2016). Comparison of collagen and gelatin extracted from the skins of Nile tilapia (Oreochromis niloticus) and channel catfish (Ictalurus punctatus). Food Bioscience..

[20] Riaz T, Zeeshan R, Zarif F, Ilyas K, Muhammad N, Safi SZ, Rahim A, Rizvi SA, Rehman IU (2018). FTIR analysis of natural and synthetic collagen. Appl Spectrosc Rev..

[21] Chen J, Li L, Yi R, Xu N, Gao R, Hong B. Extraction and characterization of acid-soluble collagen from scales and skin of tilapia (Oreochromis niloticus). LWT-Food Science Technology. 2016 Mar;1:66:453–9.

[22] Gonzalez AC, Costa TF, Andrade ZD, Medrado AR. Wound healing-A literature review. Anais brasileiros de dermatologia. 2016;91(5):614–20. 10.1590/abd1806-4841.20164741 PMC5087220 27828635

[23] Tonnesen MG, Feng X, Clark RA. Angiogenesis in wound healing. InJournal of Investigative Dermatology Symposium Proceedings 2000 Dec 1 (Vol. 5, No. 1, pp. 40–46). Elsevier. 10.1046/j.1087-0024.2000.00014.x 11147674

[24] Akita S, Akino K, Hirano A (2013). Basic fibroblast growth factor in scarless wound healing. Adv Wound Care..

[25] Atiba A, Nishimura M, Kakinuma S, Hiraoka T, Goryo M, Shimada Y, Ueno H, Uzuka Y (2011). Aloe vera oral administration accelerates acute radiation-delayed wound healing by stimulating transforming growth factor-β and fibroblast growth factor production. Am J Surg..

[26] Faler BJ, Macsata RA, Plummer D, Mishra L, Sidawy AN (2006). Transforming growth factor-β and wound healing. Perspect Vasc Surg Endovasc Ther..

[27] Penn JW, Grobbelaar AO, Rolfe KJ (2012). The role of the TGF-β family in wound healing, burns and scarring: a review. Int J Burns Trauma.

[28] Atiba A, Ueno H, Uzuka Y. The effect of aloe vera oral administration on cutaneous wound healing in type 2 diabetic rats. Journal of Veterinary Medical Science. 2011;73(5):583. 10.1292/jvms.10-0438 21178319

[29] Eming SA, Brachvogel B, Odorisio T, Koch M (2007). Regulation of angiogenesis: wound healing as a model. Prog Histochem Cytochem..

[30] Grinnell F (1994). Mini-review on the cellular mechanisms of disease. J Cell Biol.

[31] Desmoulière A, Chaponnier C, Gabbiani G (2005). Tissue repair, contraction, and the myofibroblast. Wound Repair Regen..

[32] Reza Ghassemifar M, Schultz GS, Tarnuzzer RW, Salerud G, Franzén LE. Alpha-smooth muscle actin expression in rat and mouse mesenteric wounds after transforming growth factor‐β1 treatment. Wound Repair Regen. 1997;5(4):339–47. 10.1046/j.1524-475X.1997.50408.x 16984444

[33] Pastar I, Stojadinovic O, Yin NC, Ramirez H, Nusbaum AG, Sawaya A, Patel SB, Khalid L, Isseroff RR, Tomic-Canic M (2014). Epithelialization in wound healing: a comprehensive review. Adv Wound Care..

[34] Demidova-Rice TN, Hamblin MR, Herman IM (2012). Acute and impaired wound healing: pathophysiology and current methods for drug delivery, part 2: role of growth factors in normal and pathological wound healing: therapeutic potential and methods of delivery. Adv Skin Wound Care..

[35] Dickinson LE, Gerecht S (2016). Engineered biopolymeric scaffolds for chronic wound healing. Front Physiol..

[36] Chen J, Gao K, Liu S, Wang S, Elango J, Bao B, Dong J, Liu N, Wu W (2019). Fish collagen surgical compress repairing characteristics on wound healing process in vivo. Marine Drugs.

[37] Wang J, et al. Oral administration of marine collagen peptides prepared from chum salmon *(Oncorhynchus keta)* improves wound healing following cesarean section in rats. Food Nutr Res. 2015;59:26411–1. 10.3402/fnr.v59.26411 PMC4432022 25976613

[38] Li D, Yuan Q, Yu K, Xiao T, Liu L, Dai Y, Xiong L, Zhang B, Li A (2019). Mg–Zn–Mn alloy extract induces the angiogenesis of human umbilical vein endothelial cells via FGF/FGFR signaling pathway. Biochem Biophys Res Commun..

[39] Ahluwalia A, Tarnawski S. A. (2012). Critical role of hypoxia sensor-HIF-1α in VEGF gene activation. Implications for angiogenesis and tissue injury healing. Curr Med Chem.

[40] Potaros T, Raksakulthai N, Runglerdkreangkrai J, Worawattanamateekul W (2009). Characteristics of collagen from nile tilapia (Oreochromis niloticus) skin isolated by two different methods. Nat Sci.

[41] Cheng X, Shao Z, Li C, Yu L, Raja MA, Liu C (2017). Isolation, characterization and evaluation of collagen from jellyfish Rhopilema esculentum Kishinouye for use in hemostatic applications. PloS One..

[42] Turan M, Ünver Saraydýn S, Eray Bulut H, Elagöz S, Çetinkaya Ö, Karadayi K, Canbay E, Sen M. Do vascular endothelial growth factor and basic fibroblast growth factor promote phenytoin’s wound healing effect in rat? An immunohistochemical and histopathologic study. Dermatologic Surg. 2004;30(10):1303–9. 10.1111/j.1524-4725.2004.30404.x 15458527

[43] Saber S, Khalil RM, Abdo WS, Nassif D, El-Ahwany E (2019). Olmesartan ameliorates chemically-induced ulcerative colitis in rats via modulating NFκB and Nrf-2/HO-1 signaling crosstalk. Toxicol Appl Pharmacol..

[44] Nakahara T, Hashimoto K, Hirano M, Koll M, Martin CR, Preedy VR (2003). Acute and chronic effects of alcohol exposure on skeletal muscle c-myc, p53, and Bcl-2 mRNA expression. Am J Physiol-Endocrinol Metab.

[45] Hu W, Criswell MH, Fong SL, Temm CJ, Rajashekhar G, Cornell TL, Clauss MA (2009). Differences in the temporal expression of regulatory growth factors during choroidal neovascular development. Exp Eye Res..

[46] Zhu Y, Shi B, Xu Z, Liu Y, Zhang K, Li Y, Wang H. Are TGF-β1 and bFGF Correlated with Bladder underactivity induced by bladder outlet obstruction? Urologia Internationalis. 2008;81(2):222–7. 10.1159/000144066 18758225

[47] Ljvak KJ (2001). Analysis of relative gene expression data using real time quantitative PCR and the 2^<-∆∆CT > method. Methods.

